# 

**Published:** 1970-04

**Authors:** 


The
Bristol Medico-Chirurgical Journal
(Incorporating the Medical Journal of the South-West)
Established 1883

Vol. 85 (ii) | April 1970 | No. 314

### BRISTOL MEDICO-CHIRURGICAL JOURNAL

Published Quarterly

Annual Subscription 16/- Post Free

### BRISTOL MEDICO-CHIRURGICAL SOCIETY

**President 1969-70 : Dr. F. J. W. Lewis**
**President elect : Dr. Beryl Corner**
**Honorary Secretary : Dr. I. S. Bailey, 7 Percival Road, Bristol 8**
**Honorary Treasurer : Dr. Frank Ross, Bristol Royal Infirmary, Bristol 2**

The Society was founded in 1874. In 1883 publication of this Journal began and has continued quarterly since then. During the years 1949 to 1962 it appeared under the title of " The Medical Journal of the South West ".

### THE BRISTOL MEDICO-CHIRURGICAL JOURNAL

**Editorial Committee**

| | |
|---|---|
| Editor | Dr. N. J. Brown, Southmead Hospital, Bristol |
| Assistant Editor | Mr. J. B. M. Roberts |
| Members | Mr. H. B. Griffith<br>Dr. M. B. Lennard<br>Professor R. C. Wolfinden<br>Dr. D. W. Wright<br>The Hon. Treasurer and Hon. Secretary of the Society |
| Business Manager | Dr. P. J. F. Baskett, Frenchay Hospital, Bristol |
| Secretary | Mr. A. E. S. Roberts, The Library, Medical School, University Walk, Bristol 8 |

### NOTICE TO CONTRIBUTORS

The Journal is especially concerned to provide a place for the recording of medical thought, interests, and practice in and around Bristol. It therefore welcomes articles that, as well as being of immediate interest to members, will document the local and contemporary medical scene for our successors.

Original articles are invited provided that they have not been published elsewhere. References in the text should be by author's name, with year of publication in parenthesis; they should be listed at the end in alphabetical order. Illustrations should be supplied ready for reproduction. Line drawings should be in Indian ink on firm white paper or board. The author will be informed if it is found necessary to ask him to pay for the making of blocks.

The following contributions will be especially welcome : notes of forthcoming events, meetings held, degrees conferred ,staff appointments, honours and public appointments and other local news.

Twenty-five reprints of his article will be supplied free to each contributor. Quotations for larger quantities will be sent out with proofs and must be ordered when the corrected proofs are returned.

### THE MEDICAL LIBRARY

In 1890 the Bristol Medico-Chirurgical Society founded a medical library which in 1892 was combined with that of the Bristol Medical School. This became the University of Bristol Medical Library in 1925 and an agreement in that year confirmed the privilege of Members of the Society to use the University Medical Library.

### A Selection of Recent Additions to the Medical Library

ALLAN, F. D.: Essentials of human embryology. Ed. 2. (O.U.P.) Presented by Dr. E. M. Deucher

APPLE, D.: Sociological studies of health and sickness. (McGraw-Hill)

BECKER, W. et al: Atlas of otorhinolarygology and bronchoesophagology. (Saunders)

BURNET, Sir M.: Cellular immunology. Bks. 1 and 2. (C.U.P.)

BURNET, Sir M.: Changing patterns: an atypical autobiography. (Heinemann)

CIBA FOUNDATION: Symposium on foetal autonomy. (Churchill)

CROSBY, R. M. N. and LISTON, R. A.: Reading and the dyslexic child. (Souvenir Press)

DANNENFELDT, K. H.: Leonhard Rauwolf, 16th century physician, botanist and traveller. (Harvard Univ. Pr.)

DARDENNE, M. U. and NORDMANN, J. eds.: Biochemistry of the eye. (Academic Press)

DOBSON, J.: John Hunter. (Livingstone)

EL-ZAWAHRY, M.: Skin diseases in Arabian countries. Vol. 3. (The author, Cairo)

FALCONER, I. R.: Mammalian biochemistry. (Churchill)

FARIS, R. E. L. and DUNHAM, H. W.: Mental disorders in urban areas. (Chicago Univ. Pr.)

FREEMAN, H. E. et al: Handbook of medical sociology. (Prentice Hall)

FREEMAN, T.: Psychopathology and the psychoses. (Tavistock)

FRIEDSON, E.: The hospital in modern society. (Free Press)

FURTH, H. G.: Piaget and knowledge. (Prentice Hall)

GILLESPIE, J. A. ed.: Extracranial cerebrovascular disease and its management. (Butterworths)

HAYHOE, F. G. J. and FLEMANS, R. J.: Atlas of haematological cytology. (Wolfe Medical Books)

HOLMES, W. L. et al: Drugs affecting lipid metabolism. (Plenum)

HOWELLS, J. G. ed.: Modern perspectives in international child psychiatry. (Oliver & Boyd)

HUBBLE, D.: Paediatric endocrinology. (Blackwell)

JOHNSON, P. H.: General foot care. (Wright). Presented by John Wright & Sons Ltd.

KAPLAN, E. B.: Functional and surgical anatomy of the hand. Ed. 2. (Pitman)

LANDY, M. and BRAUN, W.: Immunological tolerance. (Academic Press)

LEHMAN, H. C.: Age and achievement. (O.U.P.)

MERTON, R. et al: The student physician. (Harvard Univ. Pr.)

MISHLER, E. C. and WAXLER, N. E.: Family processes and schizophrenia. (Science House)

OSTMAN, J. ed.: Diabetes (6th Congress). (Excerpta Medica)

PIAGET, J.: Mechanisms of perception. (Routledge)

RUBIN, P.: Dynamic classification of bone dysplasias. (Yearbook Publishers)

STEVENS, B. C.: Marriage and fertility of women suffering from schizophrenia. (O.U.P.)

Exploitable molecular mechanisms and neoplasia. 22nd symposium on fundamental cancer research. (Williams & Wilkins)

TURNER, C.: Family and kinship in modern Britain. (Routledge)

WILLIAMS, J. A. and COX, A. G.: After vagtomy. (Butterworths)

### Bristol Medico-Chirurgical Society

The **Clinical Meeting** was held on 12th November, 1969, at Bristol Royal Infirmary. A demonstration of cases and exhibits was followed, after a buffet supper, by short papers and a discussion of the cases.

On 19th November, 1969, a joint meeting of the Society with the Bristol Division of the British Medical Association, the Cossham Medical Society and Galenicals was addressed by **Dr. J. M. Naish** on **"Problems of a new Medical School in Africa".** Dr. Naish had recently returned from a year as Professor of Medicine in Lagos, Nigeria.

The speaker at the third meeting of the session, held on 10th December, 1969, was **Dr. J. L. Emery,** Consultant Pathologist at Sheffield Children's Hospital, who spoke on **"Unexpected Death in Children".** After a review of the incidence of "cot deaths" in infants, Dr. Emery proceeded to outline some of the possible causes and mechanisms and gave an account of his experimental work on the possible role of various types of pillow in causing suffocation. The lecture was delightfully illustrated not only by photographs but also by drawings and cartoons from the pen of the speaker himself.

The fourth meeting was held on 14th January, 1970, when **Dr. R. Langton Hewer** spoke on **"Conquering Strokes".** It is hoped to publish this address in a future number of the Journal.

### South-West Pædiatric Club

The autumn meeting was held in Bristol on 15th and 16th November, 1969. Saturday afternoon was devoted to demonstration and discussion of clinical cases.

On Sunday the following papers were given:—

**Dr. R. F. Barbour: "A Psychiatrist at a Children's Hospital".** Dr. Barbour, until recently senior child psychiatrist, spoke of the changing spectrum of paediatric referrals, the greater turnover in fewer hours, the changing demands on consultants' time and the adverse effect these could have nowadays on children more frequently distressed than diseased. The child had to adapt too quickly to too many new faces. He made suggestions as to how this iatrogenic stress could be lessened and ended by saying that the Children's Doctor was not an Adult Doctor writ small.

**Dr. W. Schutt: Glycogen Metbalism at Birth.** Liver and muscle glycogen levels were determined at birth in thirteen anencephalic foetuses. Mean values were found to be 1.33 and 1.74 G/100G tissue respectively. There was no correlation with gestational age or between levels in liver and muscle. It was concluded that whilst these babies had had satisfactory glycogen deposition they had drawn on their liver glycogen reserves before birth.

Enzyme estimations for phosphoglucomutase, amylo-1, 6, glucosidase, acid maltase, phosphorylase and glucose-6-phosphatase were performed and the mean values were all found to be at the lower end of mean values for normal adult tissue. In the experimental animal, levels at birth are usually higher than in the adult and it is reasonable to assume that in the anencephalic the enzyme levels are depressed by the absence of pituitary hormones. Only hepatic glucose-6-phosphatase correlated with foetal maturity and one wonders whether low levels of this enzyme could account for hypoglycaemia seen in some neonates.

**Dr. R. Harris: Non-esterified fatty Acids and neonatal Hypoglycaemia.** Normal mean blood glucose and non-esterified fatty acids (NEFA) levels were estimated over the first 72 hours of life in 160 healthy newborn babies of which 60 were full-term, 20 were premature and 80 were considered to be "small for dates". The last group had lower mean blood glucose and NEFA levels at all ages studied.

Of 33 hypoglycaemic babies studied, 7 had plasma NEFA levels significantly lower than the mean at the time of the lowest blood glucose. These babies could be expected to be particularly at risk from developing hypoglycaemia, as they had lost the glucose-sparing action of the NEFA at birth.

In a further group of anoxic newborn babies, plasma NEFA and blood glucose levels were much lower in full-term babies suffering from birth asphyxia than in unasphyxiated controls but, surprisingly, the levels in asphyxiated "small for dates" babies were higher than in unasphyxiated controls. The levels in premature babies seemed little affected by asphyxia. A close study of the catecholamine responses to asphyxia in the neonatal period might help to explain these differences.

**Prof. G. Dixon: The Management of Diabetes in Pregnancy.**

**Dr. A. B. Raper:** An unstable Haemoglobin, **"Hb Bristol".** (See report of Association of Clinical Pathologists.)

The next meeting is to be held at Bournemouth on 13th and 14th June, 1970.

### Association of Clinical Pathologists Wessex Branch

A meeting was held on Saturday, 8th November, 1969, at Bristol Royal Infirmary. At the morning session the following papers were read:—

**Dr. A. P. C. H. Roome and Dr. S. K. R. Clarke** reported an outbreak of three cases of **Amoebic Meningo-encaphalitis in Children.** The first case, aged 2, developed meningitic signs and became unconscious after 6 days of upper respiratory symptoms; he died after another 16 days on a respirator. The C.S.F. was purulent and contained slowly moving amoebae which were subsequently cultured and identified as Naegleria. Treatment with amphoterracin B intravenously led to a reduction in the number of living amoebae but without clinical improvement. Necropsy revealed a soft necrotic brain showing meningo-encephalitis with some calcification; amoebae were identified with difficulty in histological sections. Case 2, brother of case 1, admitted with similar symptoms, was treated immediately with amphoterracin B and recovered after 10 days, amoebae having been cultured from C.S.F. Case 3, aged 4½, who lived next door recovered from a similar illness with the same treatment, no amoebae being isolated.

The organism Naegleria is widely distributed as a free-living form in water, soil and rotting vegetation; its cysts which are non-pathogenic can be found in dust and nose-swabs. It also exists as a flagellate. Cases of amoebic meningitis have been reported from Australia, Texas, Florida, Virginia, New Zealand and Czechoslovakia. Many cases were associated with swimming and diving in polluted water. All three children now described had played in a muddy puddle in a well-manured garden after a thunderstorm following a long period of dry hot weather. Amoebae were isolated from the puddle.

**Dr. M. Hartog** discussed **Human Growth Hormone** and described its estimation by radio-immuno-assay. The level of circulating growth hormone is increased by stress and exercise, or administration of insulin, arginine or Bovril. It is decreased by the administration of glucose and high doses of steroids (as in Cushing's syndrome). Cases of dwarfism or suspected hypopituitarism can be investigated by applying one of the above stimulants and observing whether or not there is a rise in circulating hormone. Conversely in acromegaly the expected fall in hormone level following the ingestion of glucose is not seen.

**Dr. J. Gough** discussing **Recent Developments in the Histology of Hodgkin's** disease and reviewing 96 cases recommended a classification suggested at the Rye Symposium on the "Obstacles to the Control of Hodgkin's Disease" in 1965. This was modified from Lukes, dividing cases on histological grounds into 4 groups. 1) Those with a predominance of lymphocytes and reactive histiocytes (20% of cases); this group includes Hodgkin's paragranuloma, 2) Nodular sclerosing type (15%), 3) Those with mixed cellularity (30%) and 4) Those with lymphocyte depletion (35%); this group includes "Hodgkin sarcoma". These groups corresponded with an increasing bad prognosis varying from 58% five-year survival in group 1 to only 10% in group 4.

**Dr. A. B. Raper,** reporting on a new unstable haemoglobin, **"Hb Bristol",** described a man now aged 22 who had suffered since childhood from a haemolytic anaemia with Heinz bodies in the red cells. The patient was found to possess an unstable haemoglobin not demonstrable by conventional electrophoresis. Dr. Raper reviewed the progress of knowledge of unstable haemoglobins, described the structure of the particular variety found in the present case, and showed how its properties explained the patient's symptoms.

The afternoon session was devoted to demonstrations in the laboratory.

### South Western Ophthalmological Society

Two meetings were held in Bristol during 1969, the first on 6th and 7th June (combined with the Midland Ophthalmological Society) and the second on 19th September.

At the first meeting there was the usual demonstration of cases on the Friday afternoon. This was followed by the Richardson Cross Lecture in the evening, delivered by Prof. Hans Goldmann of Berne and entitled "Biomicroscopy of the Vitreous, Fundus and Periphery". On the Saturday morning a symposium was held on the subject of Orbital Disease. The speakers were Mr. Huw Griffith, Mr. H. B. Stallard of London and Mr. A. Hirtenstein of Wolverhampton.

The second occasion was the Annual General Meeting. Again there was a demonstration and discussion of clinical cases and this was followed by a business meeting at which Mr. P. Jardine was elected President of the Society for the next two years.

### NOTES AND NEWS

The marriage took place on 5th January, 1970, of Mr. Robert Cooke, a former President of the Society, and Dr. Mavis Coutts. We convey to them both our very best wishes.

Many generations of former Bristol students will remember with affection **Mr. Joseph Dann** who was for many years senior technician in the Anatomy Department of the University. Mr. Dann who recently died at the age of 80 was a veteran member of the St. John Ambulance Brigade, with the distinction of having been made a Serving Brother of the Order by King George V. He was also a member of the R.N.V.R. in which he served in both world wars.

| | |
|---|---|
| M.R.C.P. | A. J. Buller<br>F. Higgs |
| F.F.R., R.C.S. | M. J. Gibson<br>C. L. Louisy |

### UNIVERSITY OF BRISTOL

### Examination Results and Dissertations Approved

| | |
|---|---|
| Ph.D.: | L. E. Lanyon, D. A. L. Shepherd. |
| M.D.: | R. J. Harris, M. I. V. Jayson, C. W. Jones, D. M. D. White. |
| M.B., Ch.B.: | Susan Baylis, R. Bell, M. F. Betts, Sarah J. Bodden, D. T. Bolton, B. M. Bryant, Margaret J. Freer, Judith A. Glover, M. H. P. Green, M. F. O'Dowd, D. G. Pope, R. J. Price, H. O. Subair, Paula van der Heyden, D. G. E. Wood. |

### NEWLY APPOINTED CONSULTANTS

**Dr. Duncan Chisholm,** who came to Bristol at the beginning of 1969 as Consultant Psychiatrist to Barrow and Southmead Hospitals, served in the Life Guards and read Classical Greats at Oxford before studying medicine at St. Thomas's Hospital. After house appointments there in general medicine and neurology, he trained as a psychiatrist at the Maudsley Hospital and then gained experience of psychiatric work in a general hospital at Hammersmith. His special interests have included a study of psychiatric patients with hyper-thyroidism and the psychiatric problems of patients being treated for chronic renal failure. He is married with three sons.

**Dr. Ian D. Fraser** has been appointed as Consultant Pathologist at the Regional Blood Transfusion Centre, Southmead, Bristol. He qualified in Bristol in 1957 and received his training in pathology at the United Bristol Hospitals and Southmead Hospital. He previously held appointments at Consultant Pathologist in Cheltenham (1964-1967) and in the Taunton and Yeovil Clinical area (1967-1969). He is particularly interested in haemolytic disease of the new-born and in myelo-proliferative disorders. He has four children and lives in Hambrook. His appointment is full time.

**Dr. Ronald Greenbaum,** Consultant Anaesthetist at Frenchay Hospital, Bristol, comes to us after a distinguished career in Manchester and Leeds. His researches have included oxygen transport during anaesthesia and oxygenation problems in chest injuries, fat embolus and certain poisonings. He is to be full time. He is married, has two children and lives in Westbury-on-Trym.

**Dr. Joy Harrison** entered Bristol University as an overseas student on an import licence from the Isle of Wight. After house appointments at Southmead and Frenchay Hospitals she eventually attained consultant status in the wilds of north Derbyshire where it was said that outbreaks of infection never occurred before her arrival. She returned to Frenchay Hospital as Consultant Bacteriologist in December, 1968.

Her hobbies are noisy and comprise the setting up of vibrations in attenuated feline intestines, steel wire and vocal chords. Her house is detached.

**Dr. Campbell Mackenzie** has been appointed Consultant Nephrologist to the Southmead Hospital Group and Bristol Royal Infirmary and Clinical Teacher in Renal Disease to the University.

A graduate of Cambridge and St. Bartholomew's Hospital, London, he has worked in Artificial Kidney Units since leaving the Royal Navy in 1961.

His main interest and research has been into the nutritional problems of patients with renal disease who are undergoing maintenance dialysis.

In his leisure time he enjoys golfing and sailing and as an ex-county rugby player is an enthusiastic follower of the Bristol team. With his wife and four children he will be living in Leigh Woods.

